# Effect of conditioning and test stimulus intensity on cortical excitability using triad-conditioning transcranial magnetic stimulation

**DOI:** 10.1007/s00221-020-05812-z

**Published:** 2020-04-22

**Authors:** Shady Safwat Hassan, Carlos Trenado, Tarek Ali Rageh, Alfons Schnitzler, Stefan Jun Groiss

**Affiliations:** 1 Institute of Clinical Neuroscience and Medical Psychology, Medical Faculty, Heinrich Heine University, Düsseldorf, Germany; 2 Department of Neurology, Assiut University Hospital, Assiut, Egypt; 3 Translational Neuromodulation Unit, Department of Psychology and Neurosciences, Leibniz Research Centre for Working Environment and Human Factors, Technical University Dortmund, Dortmund, Germany; 4 Department of Neurology, Medical Faculty, Heinrich Heine University, Moorenstr 5, 40225 Düsseldorf, Germany

**Keywords:** Transcranial magnetic stimulation (TMS), Motor-evoked potentials (MEP), Triad-conditioning facilitation (TCF), Conditioning stimulus (CS), Test stimulus (TS), Interstimulus intervals (ISI)

## Abstract

Cortical facilitation assessed with triad conditioning transcranial magnetic stimulation has been termed triad-conditioned facilitation (TCF). TCF has been supposed to reflect increased intracortical facilitation (ICF) at short interstimulus intervals (ISI) around 10 ms and an intrinsic rhythm of the motor cortex at longer ISI around 25 ms. To gain further insight into the pathophysiological mechanism of TCF, we systematically studied the effect of suprathreshold conditioning stimulus (CS) and test stimulus (TS) intensity on TCF. Various CS intensities and TS intensities were used in a triad-conditioning paradigm that was applied to 11 healthy subjects. ISI between pulses were studied between 5 and 200 ms. TCF at 10 ms ISI enhanced with increasing CS intensity but decreased with increasing TS intensity. The duration of facilitation was longer with higher CS intensity. However, TCF at 25 ms ISI could not be elicited with none of the CS and TS intensities addressed here. Our results are consistent with the notion of TCF at short ISI reflecting ICF. The enhanced and prolonged facilitation with increase of CS without additional isolated facilitation at longer ISI suggest a prolongation of ICF.

**Electronic supplementary material:**

The online version of this article (10.1007/s00221-020-05812-z) contains supplementary material, which is available to authorized users.

## Introduction

Transcranial magnetic stimulation (TMS) provides a sensitive and non-invasive tool to modulate excitability of different components of neural tissues (Kobayashi and Pascual-Leone 2003). Various conditioned TMS paradigms have been reported using the paired-pulse technique. For instance, a suprathreshold test stimulus (TS) preceded by a subthreshold conditioning stimulus (CS) at very short interstimulus intervals (ISI) below 5 ms gives place to suppression of MEPs (Short-interval IntraCortical Inhibition = SICI). On the other hand, when preceded by a subthreshold CS at longer ISIs, such as 10–15 ms, MEPs are facilitated (IntraCortical Facilitation = ICF) (Kujirai 1993; Ziemann 1996; Ziemann and Chapter 2003; Hanajima 2002).

Hanajima et al. reported a new triad-conditioning method to study the modulation of motor cortical excitability in response to rhythmic stimulation of M1 (Hanajima (2009)). They used three monophasic TMS pulses over M1 applied at certain frequency in terms of rhythmic conditioning stimulation and found facilitation of the MEP to TS at an ISI around 10 ms and 25 ms, which was termed triad conditioned facilitation (TCF). TCF has been supposed to reflect increased ICF at short ISI around 10 ms and intrinsic rhythm of the motor cortex at longer ISI around 25 ms. Noteworthy, alteration of TCF at 25 ms has been shown in several diseases like cortical myoclonus, Parkinson´s disease or amyotrophic lateral sclerosis (Hanajima 2011, 2014; Groiss 2017). In Hanajima’s first report (2009), the effect of varying CS intensity showed that a specific combination of CS [intensity 110% of active motor threshold (AMT), ISI = 25 m] is required to evoke TCF (Hanajima 2009). However, the effect of CS with higher intensity or variation of the TS intensity have not been investigated so far. The aim of this study was to probe the effect of variable CS and TS intensities on TCF to shed further light on its pathophysiological mechanism.

## Materials and methods

### Subjects

Eleven healthy volunteers (eight men and three women, range 22–45 years, mean 28.1, SD 7.4) participated in the study after giving written informed consent. No subject had neurological, psychiatric, or other medical problems or any contraindication to TMS (Rossi (2009)). The experiment was performed according to the Declaration of Helsinki and was approved by the Ethics Committee of the University of Düsseldorf, Germany (5738R).

### Electromyography (EMG) recordings

Surface electromyography (EMG) signals were recorded from the right first dorsal interosseous (FDI) muscle in a belly tendon montage using 9-mm diameter Ag–AgCl surface cup electrodes. Responses were amplified (Digitimer D360, UK) and filtered (100–5000 Hz), digitized at a sampling rate of 5 kHz, and stored on a computer that was used to perform the off-line analysis. Subjects were instructed to keep the right FDI relaxed throughout the experiment, which was monitored online with an oscilloscope.

### Transcranial magnetic stimulation

Magstim 200^2^ magnetic stimulators (The Magstim Company Ltd., UK) were used to deliver TMS. Four magnetic stimulators were connected with a specially designed combining module (The Magstim Co. Ltd., Whitland, UK) to allow the application of up to four monophasic magnetic stimuli through a single figure-of-eight shaped coil with an outer diameter of 7 cm at each wing (Hanajima 2009; Groiss 2017, 2013). Left M1 was chosen as stimulation site. First, the hotspot for the right FDI was identified. Subsequently, resting motor threshold (RMT) and AMT were precisely determined. RMT was defined as the lowest stimulator output intensity capable of eliciting MEPs of 50 µV peak-to-peak amplitude in the relaxed FDI muscle in more than 5 of 10 consecutive trials (Rossini et al. 2015). AMT was defined as the lowest stimulation intensity that still evoked small responses of 100 µV amplitudes in half of the trials during slight voluntary contraction (approx. 5–10% of maximal contraction) (Groiss 2017; Rothwell et al. 1999). In case of presence of a clear silent period of any duration, smaller amplitudes of 50 µV were also regarded as response.

### Triad conditioned paradigm

The original protocol of the triad conditioning paradigm consisted of a suprathreshold TS preceded by three monophasic CS with varying ISI. The intensity of the CS was set to 110% of AMT and the intensity of the TS was set to elicit MEPs of about 0.3 mV (Hanajima 2009). In this study, we performed two blocks of trials for each subject to investigate the effect of various TS and CS intensities. In the first block, the intensity of the CS was fixed to 110% AMT and we applied 3 different TS intensities according to the RMT (100% RMT, 120% RMT and 140% RMT), we named this block TS block. In the second block, we fixed the TS intensity at 120% RMT and used variable CS intensities according to the AMT (110% AMT, 120% AMT and 130% AMT), so this block was named CS block. The ISIs were varied between 5 and 200 ms resulting in 10 conditions (5, 10, 12.5, 20, 25, 40, 50, 100, 200 ms which corresponds to the following frequencies in order (200, 100, 80, 66, 50, 40, 25, 20, 10, 5 Hz) and one control condition (for the test stimulus alone) and each condition was applied in a shuffled randomized order. Compared to the original report, the number of conditions were reduced to allow the measurements to be done in one session.

### Statistical and data analysis

Statistical analysis was performed using GraphPad Prism (GraphPad Software, CA, USA) and IBM SPSS Statistics (Version 24, IBM Software, Business and analytics, Armonk, NY, USA). We used two-way repeated measures ANOVA with within-factor ISI and between factor AMT- and RMT- intensities for each block to compare triad-conditioned MEPs. Post hoc Bonferroni tests were performed whenever an interaction was found. One-way ANOVA with post hoc Dunnett’s test was done in each group to compare the degree of facilitation at each ISI with the baseline. To determine a prospective relationship between the degree of facilitation at each ISI and MEP amplitudes at TS intensity (MSO%), correlation analysis with the Holm correction for multiple comparisons was performed [by considering parameters $m=18$ (number of *p* values) and threshold *p = 0.003 similarly as specified in (Trenado et al. 2018)].

## Results

**Fig. 1:**
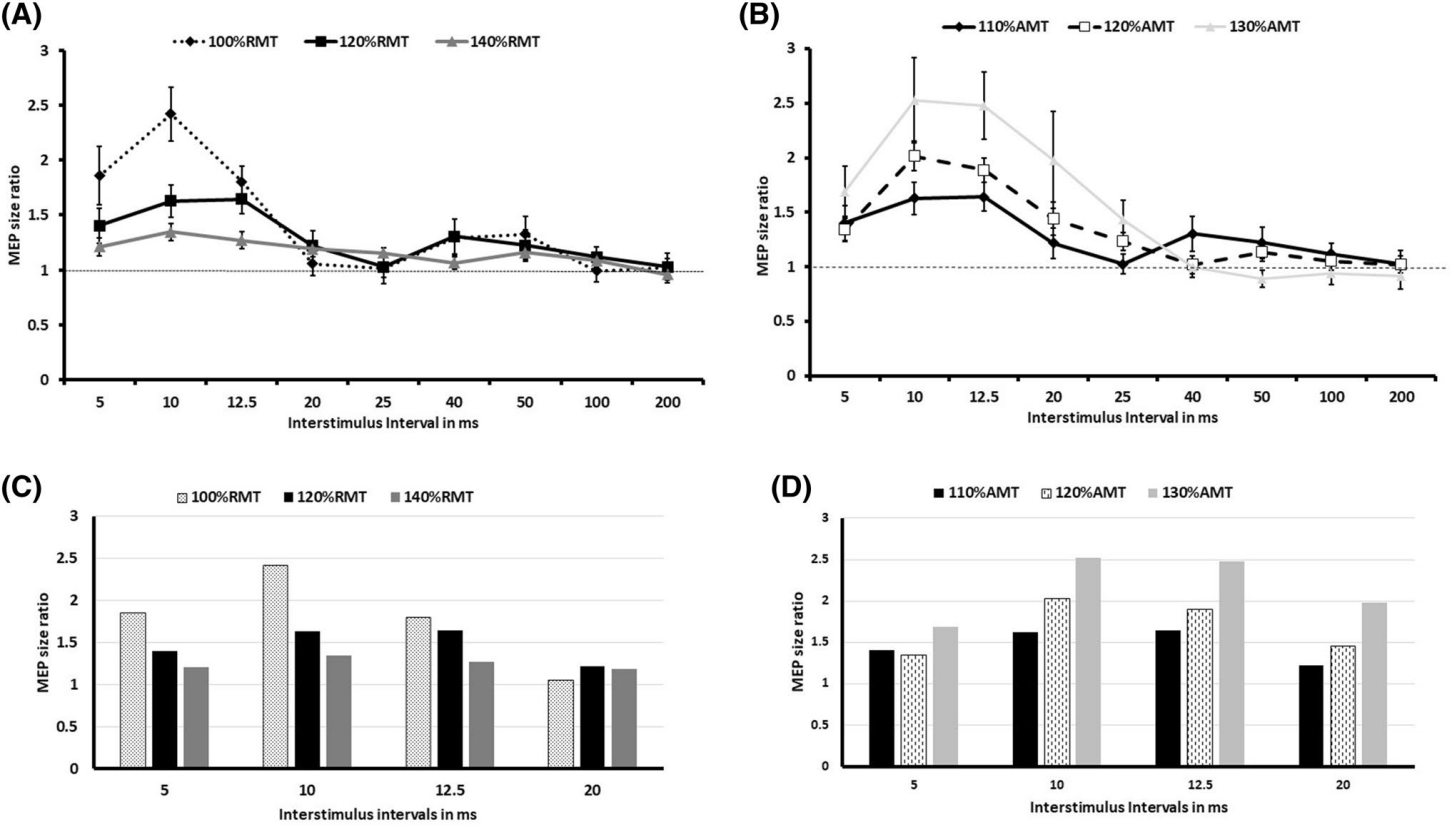
** a** TS block results. Here, we show TCF results across ISIs under by varying of the test stimulus intensities; **b** CS block results. Here, it is shown TCF results across ISISs by varying intensities of the conditioning stimuli; **c**, **d** display bar graphs describing the comparison between different TS and CS intensities in the ISI between 5 and 20 ms

**Fig. 2:**
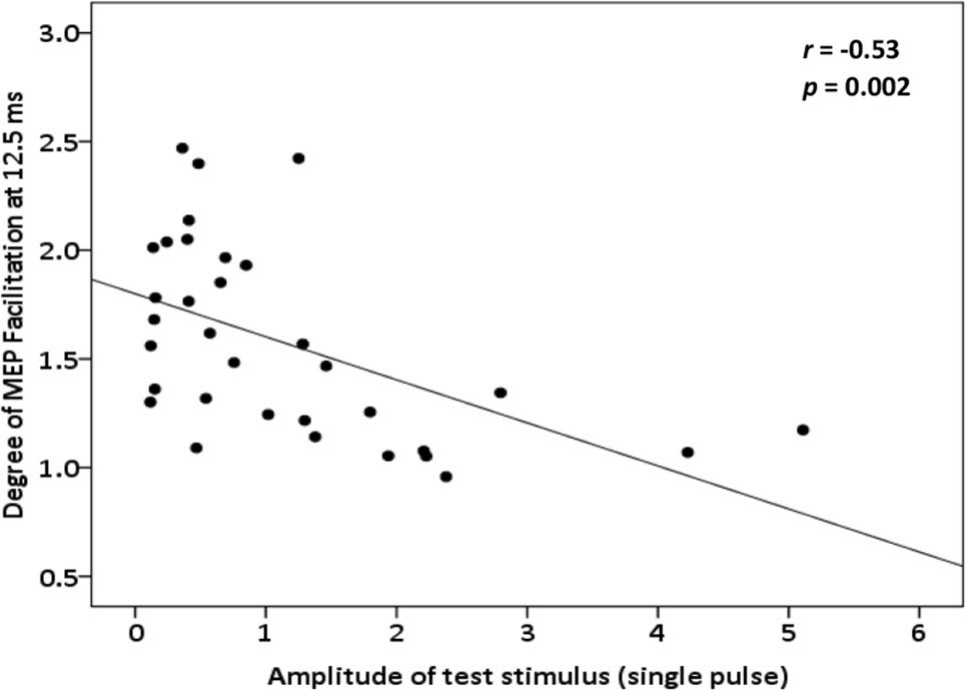
Correlation analysis between MEP amplitude at TS intensity and degree of facilitation at 12.5 ms. Insets illustrate the results of the linear correlation analysis (black line)

Figure 1a, b shows MEP size ratios as a function of ISI for the different TS and CS intensity settings. Two-way repeated measures ANOVA showed significant effect of ISI and interaction between ISI and stimulus intensity for both blocks. For TS block [ISI: *F* (8, 240) = 21.75, *p* < 0.0001; test intensity: *F* (2,240) = 2.19, *p* = 0.12; interaction (ISI × test intensity): *F* (16, 240) = 4.7, *p* < 0.0001], post hoc Bonferroni test revealed significant differences in MEP amplitudes between TS intensities 100% RMT and 120% RMT at ISI 10 ms and between 100% RMT and 140% RMT at ISI 5, 10 and 12.5 ms. For CS block [ISI: *F* (8, 240) = 27.9, *p* < 0.0001; test intensity: *F* (2,240) = 1.16, *p* = 0.37; interaction (ISI × test intensity): *F* (16, 240) = 3.72, *p* < 0.0001], post hoc Bonferroni test showed significant differences in MEP amplitudes between CS intensity 110% AMT and 130% AMT at ISI 10, 12.5 and 20 ms (Fig. 1c, d). Compared to baseline one-way ANOVA with post hoc Dunnett’s test revealed the following results: For 100% RMT intensity there was significant MEP facilitation at 5, 10, 12.5 ms, while in 120% RMT and 140% RMT facilitation was significant at 10 and 12.5 ms. In the CS block there was significant facilitation for 110% AMT at 10 and 12.5 ms and for 120% AMT and 130% AMT at 10, 12.5 and 20 ms in comparison to the baseline. Figure 2 shows significant negative correlation between degree of MEP facilitation at 12.5 ms and single pulse MEP amplitude (*r* = − 0.53, *p* = 0.002).

The following TMS characteristics were found in the current study: Thresholds for the study participants [RMT (54.3% ± 8.3), AMT (35.5 ± 5.8)]; unconditioned MEP amplitudes for each TS intensity (100% RMT, 120% RMT and 140% RMT) across the different CS conditions were as follows: 0.3, 1.01 and 2.1 mV, respectively. It is worth to emphasize that a TS intensity of 100% RMT in our study was the most comparable condition to the criterion set by Hanajima et al. of using a TS intensity of 0.3 mV.

## Discussion

In this study, we confirmed two main results. First, the triad conditioning TMS induced MEP facilitation at 10 and 12.5 ms that was enhanced with increasing CS intensity but decreased with increasing TS intensity, which supports the notion of TCF at short ISI sharing the same mechanisms as ICF (Kujirai 1993; Daskalakis 2004). Second, the duration of TCF at shorter ISI became longer as CS intensity increased but isolated TCF at 25 ms was lacking with the addressed intensities here. This suggest a prolongation of ICF with CS intensity increase rather than a potentiation of intrinsic cortical rhythms.

In our study, when we increased intensities of CS (110%AMT, 120%AMT and 130%AMT) preceding a 120% RMT TS intensity, a gradual increase of facilitation occurred at short intervals around 10 ms and lasting up to 20 ms with lack of facilitation at 25 ms. So, we found highest facilitation using the maximum conditioning subthreshold intensity (130%AMT). On the other hand, facilitation decreased when we used a gradually increased intensity of the test stimulus following a CS at 110%AMT. In summary, facilitation was enhanced with increasing CS intensity and reduced with increasing TS intensity. This could be explained by the fact that higher intensity of TS may result in larger recruitment of interneurons making synaptic connections to corticospinal neurons and lower threshold interneurons responding with weaker intensity. Thus, reduction of TCF with higher intensity of TS might indicate less influence of conditioning stimuli. Accordingly, this will be in line with intensity dependent changes of ICF which have been described in paired-pulse TMS paradigms and, therefore, supports the hypothesis of TCF around 10 ms sharing the same mechanism as ICF (Kujirai 1993; Ziemann and Chapter 2003). These findings were confirmed by the correlation analysis, where the degree of facilitation at 12.5 ms ISI inversely correlated with the MEP amplitude at TS intensity. At the same time, we noticed prolonged duration of facilitation with increased intensities of both CS and TS.

The original experiments designed by Hanajima et al. (2009), reported incidence of MEP facilitation at two peaks. The first peak at ISI around 10 ms which is compatible with ICF and the second termed TCF and occurring at ISI 25 ms which is suggested to represent increased motor cortical responsiveness to rhythmic external stimuli that is induced by an intrinsic 40 Hz rhythm of the motor cortex (Hanajima 2009). Owing to the relevance of the TCF at 25 ms for differentiation or diagnostics of neurological diseases as reported by previous clinical studies, we aimed to probe the effect of variable CS and TS intensities on TCF to shed further light on its pathophysiological mechanism. Interestingly, TCF at 25 ms was not elicited in our case. Several possibilities for explanation need to be taken into account. Given the fact that duration of ICF (in terms of TCF at short ISI) was prolonged by increasing CS without additional isolated TCF at longer ISI, facilitation at 25 ms may also reflect prolongated ICF, which would argue against the rhythm hypothesis for TCF 25 ms. However, it is crucial to emphasize that there were methodological differences regarding our TMS settings and the previous studies. First of all, in the original paper, a coil orientation to induce PA currents in the brain with the coil handle in parallel to the midline was used. In our case, we also used PA currents but with coil handle oriented 45 degrees from the midline. The relevance of this aspect has been emphasized by previous studies reporting on changes in amplitude of MEP output as a function of coil orientation (Mills et al. 1992). However, since we were able to induce TCF 25 ms in our previously published paper, where we used the same current direction, the coil orientation may less likely be the reason for the differing results (Groiss et al. 2017). However, we believe that another prospective analysis comparing different coil directions will be necessary in the future to clarify this point.

Second, in the original reports, TS intensity was chosen based on the MEP amplitude (the intensity which elicit MEPs of about 0.3 mv when given alone) and fixed the CS intensity to 110% AMT (Hanajima et al. 2009). In our study we adjusted our TS intensity referring to RMT and not to the MEP amplitude. However, single pulse TMS in the 100% RMT condition in fact elicited MEP responses of 0.27 mV in average after omitting the trials without response in our study. This makes this condition likely comparable with the original reports. Finally, anatomical differences (e.g., the thickness and shape of skull) between participants in our study (which mainly considered subjects of European descent) and the previous studies, which considered Asian participants may be of relevance. Thus, ethnicity may represent a contributing factor to variability of cortical excitability in different populations due to anatomical differences as emphasized by earlier studies (Ball 2010).

Limitations of all studies on TCF include the rather small sample sizes, which further motivates multicenter studies to better understand the variability of TCF in healthy populations.

## Conclusions

Taken together, facilitation around 10 ms enhances with increasing CS intensity but decreases with increasing TS intensity. However, increasing CS intensity prolongs the duration of facilitation, while TCF at 25 ms could not be elicited. Our results are consistent with the notion of TCF at short ISI reflecting ICF. The increased duration of ICF with increase of CS without isolated TCF at longer ISI suggest a prolongation of ICF for TCF 25 ms as well and speaks against the rhythm hypothesis. The present results question the currently assumed hypothesis on TCF mechanisms and may be relevant regarding the optimization of triad conditioning paradigms for future clinical studies.

## Electronic supplementary material

Below is the link to the electronic supplementary material.

Supplementary file1 (DOCX 34 kb)
