# Supplementary figures and images for: Effect of conditioning and test stimulus intensity on cortical excitability using triad-conditioning transcranial magnetic stimulation
Source: Exp Brain Res. 2020 Apr 22;238(5):1305–9. doi: 10.1007/s00221-020-05812-z (PMC7237525; doi:10.1007/s00221-020-05812-z)

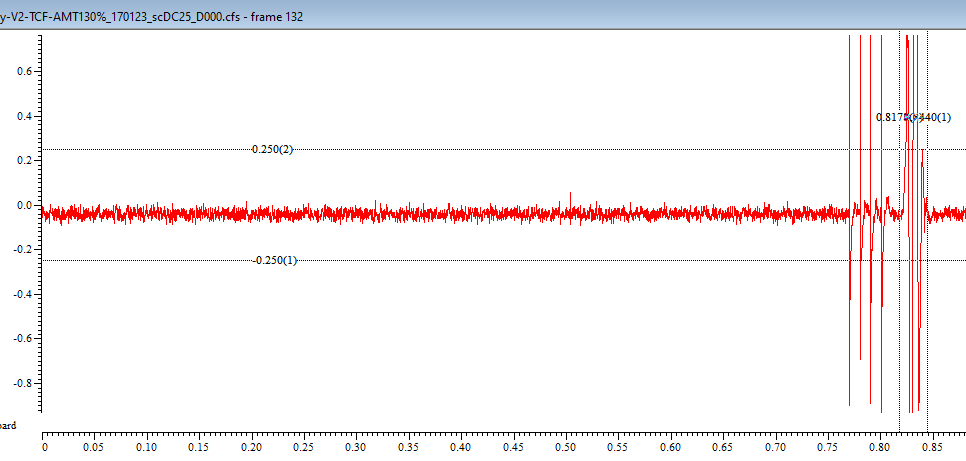

Supplement: Supplementary file 1 — Supplementary file1 (DOCX 34 kb) [file 221_2020_5812_MOESM1_ESM.docx]
